# Personalized prediction of chemotherapy efficacy in osteosarcoma through patient-derived organoids: correlation with survival and tumor proliferation potential

**DOI:** 10.1186/s13046-025-03541-1

**Published:** 2025-12-11

**Authors:** Jun-Hua Nie, Chen-Yang Wan, Hong Li, Jie-Long Zhou, Guo-Qing Zhong, Tao Yang, Meng-Yu Yao, Wen-Han Huang, Chi Zhang, Sheng Li, Jia Liu, Wei Li, Yu Zhang

**Affiliations:** 1 Guangdong Cardiovascular Institute, Guangdong Provincial People’s Hospital Ganzhou Hospital, Guangdong Academy of Medical Sciences, Ganzhou, 341000 Jiangxi China; 2 Guangdong Cardiovascular Institute, Guangdong Provincial People’s Hospital, Guangdong Academy of Medical Sciences, Guangzhou, 510030 Guangdong China; 3 School of Medicine, South China University of Technology, Guangzhou, 510006 Guangdong China; 4 BioMed Laboratory, Jingke Biotechnology Group, Bioisland International, Guangzhou, 510005 Guangdong China; 5 Liaoning Laboratory of Cancer Genetics and Epigenetics, College of Basic Medical Sciences, Dalian Medical University, Dalian, 116044 Liaoning China

**Keywords:** Osteosarcoma, Patient-derived organoids, Chemotherapy response, Drug sensitivity testing, Organoid formation, Precision medicine, Predictive biomarker

## Abstract

**Background:**

Neoadjuvant chemotherapy (NAT) is the standard treatment for osteosarcoma (OS), but patient responses vary, and conventional imaging or pathology offers limited predictive accuracy. Patient-derived organoids (PDOs) are promising models for assessing drug sensitivity and tumor viability ex vivo. This study evaluated the potential of PDO-based drug sensitivity testing and organoid formation potential (OFP) to predict therapeutic outcomes in OS.

**Methods:**

Tumor samples from OS patients collected before and after NAT were cultured as 3D PDOs. Chemosensitivity to first-line agents was quantified via a cell inhibition weighted score (CIWS), whereas OFP was used to reflect residual tumor viability. Clinical response was assessed via RECIST 1.1 and, for the primary analysis, dichotomized as responder (CR+PR+SD) versus nonresponder (PD); survival (OS/DFS) was tracked for up to 5 years. Correlations between PDO metrics and clinical outcomes were analyzed.

**Results:**

PDOs were established from 31 samples (18 pre-NAT samples and 13 post-NAT samples), including 8 paired pre/post-NAT samples. CIWS predicted NAT response assessed by RECIST 1.1 with 83.3% accuracy (pre-NAT, 15/18; 95% CI 58.6–96.4) and 5-year DFS with 84.6% accuracy (post-NAT, 11/13; 95% CI 54.6–98.1). With predefined CIWS/OFP cutoffs, Kaplan–Meier analyses showed longer DFS/OS in CIWS-sensitive and OFP-II/III (low growth) groups (*P*<0.05).

**Conclusions:**

PDOs demonstrated promise as an ex vivo approach to evaluate whether ex vivo chemosensitivity and residual tumor viability measured by PDOs are associated with imaging response and 5-year survival. Their correlation with clinical outcomes highlights the potential of PDO testing to complement existing evaluation methods and to inform individualized treatment strategies.

## Highlights

• Patient-derived organoids (PDOs) were successfully established from 31 osteosarcoma samples (18 pre- and 13 post-neoadjuvant chemotherapies) collected from 23 patients.

• PDO-based drug sensitivity and organoid formation potential reflect tumor viability and resistance.

• CIWS based on PDOs achieved 84.6% (11/13) accuracy, outperforming RECIST evaluation.

• CIWS and OFP were significantly associated with 5-year DFS and OS.

## Introduction

Osteosarcoma (OS) is the most common primary malignant bone tumor in children and adolescents, with an annual incidence of approximately 3 per million worldwide and a peak incidence during the second decade of life [1, 2]. Despite aggressive multimodal therapy, including neoadjuvant chemotherapy (NAT), surgical resection, and adjuvant chemotherapy, the 5-year survival rate for localized OS remains at 60–70%, whereas metastatic or recurrent disease has a dismal prognosis with less than 30% long-term survival [3–5]. Although the introduction of the MAP regimen—comprising high-dose methotrexate, doxorubicin, and cisplatin—marked a therapeutic breakthrough in the 1980 s, minimal advancements in front-line OS therapy have been made in the past three decades [4, 6]. A major contributor to treatment failure is the emergence of intrinsic or acquired chemoresistance, which underscores the urgent need for functional predictive models to identify responders and nonresponders before and after NAT [7, 8].

Conventional methods for evaluating treatment efficacy, such as RECIST-based radiological imaging, histological tumor necrosis grading, and clinical follow-up, have limited temporal and biological resolution [9, 10]. Morphological imaging may underestimate early signs of therapeutic failure, and pathological necrosis—although prognostically informative—is a retrospective indicator requiring tumor resection, thus offering no real-time therapeutic guidance [11]. Moreover, recent studies have highlighted that the histological heterogeneity of OS and the dynamic evolution of drug resistance under chemotherapy pressure cannot be reliably captured by static assessments [12, 13]. Therefore, functional models that can simulate in vivo tumor behavior ex vivo and provide timely drug sensitivity data are of paramount importance in the management of OS.

Three-dimensional (3D) patient-derived organoids (PDOs) have emerged as powerful platforms that bridge the gap between conventional in vitro models and in vivo tumors. PDOs recapitulate the histological, genetic, and pharmacological profiles of the parent tumor while allowing for rapid expansion and high-throughput drug screening [14–16]. Compared with standard biomarkers or imaging, PDO-based drug sensitivity assays have demonstrated superior predictive value for clinical response in epithelial tumors such as colorectal, pancreatic, and lung cancers [17, 18]. However, the application of PDOs in sarcomas—including osteosarcoma—has been limited due to technical challenges in 3D culture initiation, the low proliferative capacity of postchemotherapy residual tumors, and the lack of validated functional[19]. However, recent advancements in sarcoma-specific culture systems and the use of functional proliferation and viability metrics have enabled the generation of OS organoids that mirror patient-specific drug response patterns [20].

This study establishes OS PDOs as a clinically actionable theranostic tool capable of predicting the response to NAT and long-term outcomes in osteosarcoma patients. By integrating PDO-based drug screening with organoid viability metrics, we provide a functional precision oncology framework for real-time decision-making in OS management. Our findings lay the groundwork for incorporating PDO platforms into clinical trial design, risk stratification, and treatment customization in osteosarcoma patients.

## Methods

**Patient eligibility**

All experiments were performed in accordance with institutional ethical guidelines and approved by the Ethics Committee of Guangdong Provincial People’s Hospital, Guangdong Academy of Medical Sciences (Approval No. GDREC2020093A). Written informed consent was obtained from each patient prior to sample collection. Eligible patients with a confirmed diagnosis of osteosarcoma were enrolled between January 2020 and December 2020.

**Sample collection and classification**

Resected osteosarcoma specimens were obtained from the Department of Bone Oncology and transferred to the laboratory within one hour of surgical resection. A total of 23 tumor samples were collected, comprising 20 osteoblastic osteosarcomas and 3 chondroblastic osteosarcomas. Each fresh sample was dissected on ice and minced into small fragments. A portion of the tumor tissue was snap-frozen in liquid nitrogen and stored at –80 °C for future molecular analyses. The remaining tissue was subjected to standard pathological evaluation: sections were examined both grossly and microscopically, and the histopathological subtype and grade were confirmed by board-certified pathologists according to established osteosarcoma classification criteria.

**Organoid culture**

The tumor tissue fragments were first rinsed thoroughly (five times) in chilled transport medium consisting of Advanced DMEM/F12 (Gibco, #12634010, NY, USA) supplemented with 10% fetal bovine serum (FBS; Gibco, #10099141, Australia), penicillin (500 U/mL) and streptomycin (500 µg/mL) (Gibco, #15070063, Canada), and nystatin (50 µg/mL; Sangon Biotech, #89104730, Shanghai, China). The tissue was then transferred to a sterile 3-cm Petri dish (Jet Biofil Tech, #TCD000035, Guangzhou, China) and mechanically minced into fragments smaller than 0.1 mm in diameter via sterile scalpels.

The minced tissue was enzymatically digested in Dulbecco’s modified Eagle’s medium (DMEM) containing 10% FBS and TrypLE Express enzyme ($1\times$, no phenol red; Gibco, #12604021, NY, USA) at 37 °C for 90 min. The digestion was quenched by adding cold PBS with 10% FBS, and the cells were collected by centrifugation. The resulting single-cell suspension was mixed 1:2 (v/v) with growth factor–reduced Matrigel (Corning, MA, USA). Aliquots of 10,000–20,000 cells in 30 µL of the Matrigel mixture were seeded as droplets in 48-well culture plates. The Matrigel droplets were allowed to solidify at 37 °C for 30 min before overlaying each well with 300 µL of organoid culture medium.

The organoid culture medium consisted of Advanced DMEM/F12 (Gibco, #12634010) supplemented with GlutaMAX ($1\times$, Gibco, #35050061), penicillin (100 U/mL) and streptomycin (100 µg/mL) (Gibco, #15070063), nystatin (20 µg/mL; Sangon Biotech, #89104730), N-acetylcysteine (1 mM; Sigma–Aldrich, #106425, St. Louis, MO, USA), A83-01 TGF-$\upbeta$ inhibitor (0.5 µM; Tocris, #909910, Bristol, UK), B-27 supplement minus vitamin A ($1\times$; Invitrogen, #12587010, Waltham, MA, USA), epidermal growth factor (EGF, 50 ng/mL; PeproTech, #AF-100-15, NJ, USA), R-spondin-1 (RSPO1, 500 ng/mL; PeproTech, #120-38), the p38 MAPK inhibitor SB202190 (10 µM; Sigma–Aldrich, #152121), the ROCK inhibitor Y-27632 (10 µM; Sigma–Aldrich, #129830-38-2), and Cultures were maintained in a 37 °C humidified incubator with 5% CO_2_, and the medium was changed every 2–3 days. Organoids typically form within 2–3 weeks of culture. After 14 days of growth, the organoids were either harvested for assays or cryopreserved for storage. For long-term preservation, the organoids were resuspended in CryoStor CS10 cryoprotectant (Stemcell Technologies, #07930, Vancouver, Canada), gradually cooled, and stored in liquid nitrogen.

**Immunofluorescence and H&E staining**

For histological and immunofluorescence analysis, organoid cultures were harvested from the Matrigel and embedded in agarose prior to sectioning. Organoids were released from the Matrigel matrix by incubating the culture drops with chilled Cell Recovery Solution (Corning, #CLS354253, MA, USA) on ice for 30 min. The organoids were collected by gentle centrifugation (1,000 rpm for 5 min) and fixed in 4% paraformaldehyde for 1 h at room temperature. After fixation and PBS washes, the organoid pellets were embedded in 2% low-melting-point agarose. Once solidified, the agarose-encased pellets were processed into paraffin blocks via standard histological procedures. The paraffin-embedded organoids were then cut into 5 µm sections with a microtome and mounted on slides for staining.

For IF staining, deparaffinized and rehydrated sections were subjected to heat-induced antigen retrieval in citrate buffer (pH 9.0; OriGene, #ZLI-9069; Beijing, China) via a steam pressure cooker. The sections were cooled and then blocked for 20 min at room temperature in PBS containing 0.1% bovine serum albumin (BSA), 0.3% Triton X-100, and 0.05% Tween-20 to prevent nonspecific binding. Primary antibodies, including anti-SOX9 (1:1000 dilution; Abcam, #ab185966, Cambridge, UK) and anti-vimentin (1:1000; Abcam, #ab8069), were applied overnight at 4 °C. After thorough washing in PBS, the slides were incubated with appropriate fluorophore-conjugated secondary antibodies (against rabbit and mouse IgG) for 1 h at room temperature in the dark. Nuclei were counterstained with 4’,6-diamidino-2-phenylindole (DAPI), and coverslips were mounted with antifade medium. Stained organoid sections were visualized and imaged via a fluorescence microscope ($200\times$ magnification).

For hematoxylin and eosin (H&E) staining, paraffin sections were dewaxed, rehydrated through a graded alcohol series, and stained via a commercial H&E kit (Solarbio, #G1120, Beijing, China) according to the manufacturer’s protocol. After sequential hematoxylin and eosin staining, the slides were dehydrated, cleared, and coverslipped. The morphological features of the organoids were examined under a light microscope.

**Organoid formation evaluation**

The process of organoid formation in culture was monitored and quantified to assess the proliferative capacity of viable tumor cells ex vivo. Organoid growth was observed daily under a phase-contrast microscope (Nikon Eclipse Ts2, Tokyo, Japan) for up to 4 weeks. The time to initial organoid emergence varied between samples, ranging from approximately 5 days to 27 days after seeding. To characterize this variability, an **OFP** score was assigned to each sample, integrating the timing of organoid appearance and the extent of cellular proliferation (as evidenced by EdU incorporation in parallel assays). We defined three levels of OFP [21]:

- **OFP-I (Robust-forming)**: Organoids developed rapidly, appeared within the first 3 weeks of culture, and exhibited vigorous expansion with a well-defined spheroid morphology. These cultures originated from dense, highly viable cell clusters (diameter $> 100$ µm) and presented abundant EdU-positive nuclei, indicating high proliferative activity.
- **OFP-II (Intermediate-forming)**: Organoids formed and remained viable after 3 weeks, but the growth rate and proliferative activity gradually declined over time. These organoids typically arose from smaller tumor cell clusters (80 µm in diameter) and demonstrated moderate levels of EdU incorporation.
- **OFP-III (Minimal/Nonforming)**: Little to no organoid formation was observed after 3 weeks. Cultures in this category contained only sparse, tiny cell aggregates or necrotic debris with no significant organoid growth. Few or no EdU-positive nuclei were detected, reflecting the severely compromised proliferative capacity of the tumor cells in vitro.

**Drug sensitivity assays**

Drug sensitivity testing of the osteosarcoma organoids was performed via five first-line chemotherapeutic agents commonly employed in osteosarcoma treatment. These included doxorubicin, cisplatin, methotrexate, ifosfamide, and carboplatin, all of which were obtained from MedChemExpress (HY-15142A, HY-17394, HY-14519, HY-17419, and HY-17393; Monmouth Junction, NJ, USA). Stock solutions for each drug were prepared in dimethyl sulfoxide (DMSO) and diluted in organoid culture medium to the following working concentrations: 1 µM doxorubicin, 4 µM cisplatin, 10 µM methotrexate, 38.3 µM ifosfamide, and 10 µM carboplatin. Osteosarcoma organoids embedded in Matrigel (in 48-well plates) were incubated in medium containing each drug for 96 h. Untreated organoids maintained in parallel served as negative controls. All the experimental conditions were set up in triplicate.

After drug exposure, organoid viability was evaluated via a live/dead cell staining assay with calcein-AM and propidium iodide (PI) to distinguish live (calcein-positive) and dead (PI-positive) cells. For each treatment group and control, at least 30 organoids (approximately 100 µm in diameter each) were analyzed to determine the fraction of nonviable cells. The cell inhibition rate (CIR) of each drug was calculated as the percentage of dead cells in the drug-treated organoids. A standardized cell inhibition rate (SCIR) was then derived for each drug by subtracting spontaneous cell death in control organoids: SCIR = CIR_drug – CIR_control. This SCIR represents the increase in cell killing attributable to the chemical agent.

On the basis of the SCIR values, drug responses in the organoids were categorized into qualitative efficacy tiers as follows:

- Not effective (–): SCIR $< 30$%
- Possibly effective (+): SCIR 30% to $< 60$%
- Most likely, effective (++): SCIR 60% to $< 80$%
- Highly effective (+++): SCIR $\ge$ 80%

To predict overall chemosensitivity to combination therapy, we further calculated a composite score termed the cell inhibition weighted score (CIWS). The CIWS was obtained by summing the efficacy grades of the four core drugs in the osteosarcoma regimen (doxorubicin, cisplatin, methotrexate, and ifosfamide, collectively referred to as the MAPI regimen). For example, a “+++” rating was assigned a value of 3, “++” a value of 2, “+” a value of 1, and “–” a value of 0; the grades from the four drugs were added together. Following Liu et al. [22], we derived dichotomization thresholds via maximally selected rank statistics (R packages maxstat and survminer::surv_cutpoint) on the basis of disease-free survival (DFS) and overall survival (OS). To enhance interpretability and generalizability, candidate cutoff points were restricted to ordinal midpoints (e.g., 3.5, 4.5, and 5.5), a minimum group proportion was enforced (minprop = 0.25), and stability was assessed via bootstrap resampling (B = 1,000). Among the discrete candidates, 4.5 emerged as the survival-optimal midpoint for both DFS and OS (statistically significant). The integer-rounding rule corresponds to CIWS $\ge$ 5. We therefore selected CIWS $\ge$ 5+ as the drug sensitivity cutoff. Using the same statistical framework, we contrasted OFP-I vs. OFP-II/III to stratify tumor growth potential, designating OFP-I as the high–growth-potential group and OFP-II/III as the low–growth-potential group. This approach achieved the highest concordance with clinical outcomes in our internal validation.

**Clinical outcome assessment**

The clinical outcomes of the corresponding patients were compared with the organoid-based predictions. The radiological tumor response after neoadjuvant chemotherapy was evaluated in each patient via the Response Evaluation Criteria in Solid Tumors (RECIST 1.1). On the basis of imaging measurements, patients were classified as having a complete response (CR), partial response (PR), stable disease (SD), or progressive disease (PD). Per RECIST v1.1, CR is the disappearance of all target lesions and any pathologic lymph nodes with a short axis $<10$ mm; PR is a $\ge$30% decrease from baseline in the sum of longest diameters (SLD); SD denotes neither sufficient shrinkage for PR nor increase for PD; PD is a $\ge$20% increase in SLD from the nadir with an absolute rise $\ge$5 mm or the appearance of new lesions or unequivocal progression of nontarget disease [23]. After surgical resection and completion of all planned therapy, patients were followed for disease recurrence and survival. Disease-free survival (DFS) was defined as the time from the initial date of definitive surgery to the first occurrence of relapse or progression (or to the last follow-up if no relapse occurred). Given that the majority of osteosarcoma relapses occur within 5 Years of diagnosis, a 5-year follow-up was used as a benchmark for long-term outcomes. Patients who experienced a relapse within less than 5 years were considered to have chemoresistant disease (poor responders to therapy), whereas those who remained disease-free at 5 years were considered to have had a good response (chemosensitive disease).

**Accuracy calculation**

For each analysis in which predictive accuracy was reported, we explicitly defined both the prediction output and the reference ground truth. (1) For pre-NAT PDOs, predictions were based on the binarized CIWS score (sensitive if CIWS $\ge$ 5+, resistant if CIWS $< 5$+) and were compared against the post-NAT radiological response by RECIST 1.1. Here, we defined RECIST-NAT as the radiological response assessed by RECIST 1.1 immediately after the completion of NAT, dichotomized as responder (CR/PR/SD) versus nonresponder (PD). AT-RECIST (5y) was defined as RECIST 1.1 assessed 5 years after curative-intent surgery, in patients who completed NAT and adjuvant therapy; responses were dichotomized as responder (CR/PR/SD) versus non-responder (PD). For consistency, RECIST responses were binarized as responder (CR + PR + SD) vs nonresponder (PD). (2) For post-NAT PDOs, predictions were again based on CIWS binarization (sensitive vs resistant) and were compared against 5-year disease-free survival (DFS), which was dichotomized as disease-free at 5 years (no recurrence or death) versus recurrence within 5 years. (3) For RECIST-based predictions, RECIST classification after NAT was dichotomized (responder vs nonresponder) and compared with the 5-year DFS outcome. The predictive accuracy was then calculated as the proportion of correctly classified patients relative to the corresponding ground truth label in each comparison. In this context, the “PDO vs RECIST-NAT” row indicates the concordance between PDO CIWS-based predictions and the RECIST assessment after neoadjuvant therapy (NAT). The “PDO vs RECIST-AT (5y)” row indicates the concordance between the PDO CIWS-based predictions and the RECIST assessment at 5 years after curative-intent surgery. The predictive accuracy was calculated as the proportion of correctly classified patients relative to the corresponding ground truth label in each comparison.

In this context, the “PDO vs RECIST-NAT” row indicates the concordance between PDO CIWS-based predictions and the RECIST assessment after neoadjuvant therapy (NAT). The “PDO vs RECIST-AT (5y)” row indicates the concordance between the PDO CIWS-based predictions and the RECIST assessment at 5 years after curative-intent surgery. The predictive accuracy was calculated as the proportion of correctly classified patients relative to the corresponding ground truth label in each comparison.

**Statistical analysis**

Statistical analyses were performed using R (v4.x) and SPSS (v26). CIWS and OFP thresholds were determined via maximally selected rank statistics with 1,000-bootstrap validation. Survival analyses employed Kaplan-Meier curves with log-rank tests and Cox regression. Predictive accuracy metrics (sensitivity, specificity, PPV, NPV) were calculated with exact 95% CIs. Agreement was assessed using Cohen’s $\upkappa$ and McNemar tests. Correlations were evaluated using Spearman’s $\uprho$ and Kendall’s $\uptau$-b. Model performance was assessed via C-index, time-dependent AUC, and decision curve analysis. Group comparisons utilized Wilcoxon, $\upchi ^{2}$, or Fisher’s exact tests as appropriate. Two-tailed $P<0.05$ was considered significant.

## Results

**Patient** **demographics**

**Fig. 1:**
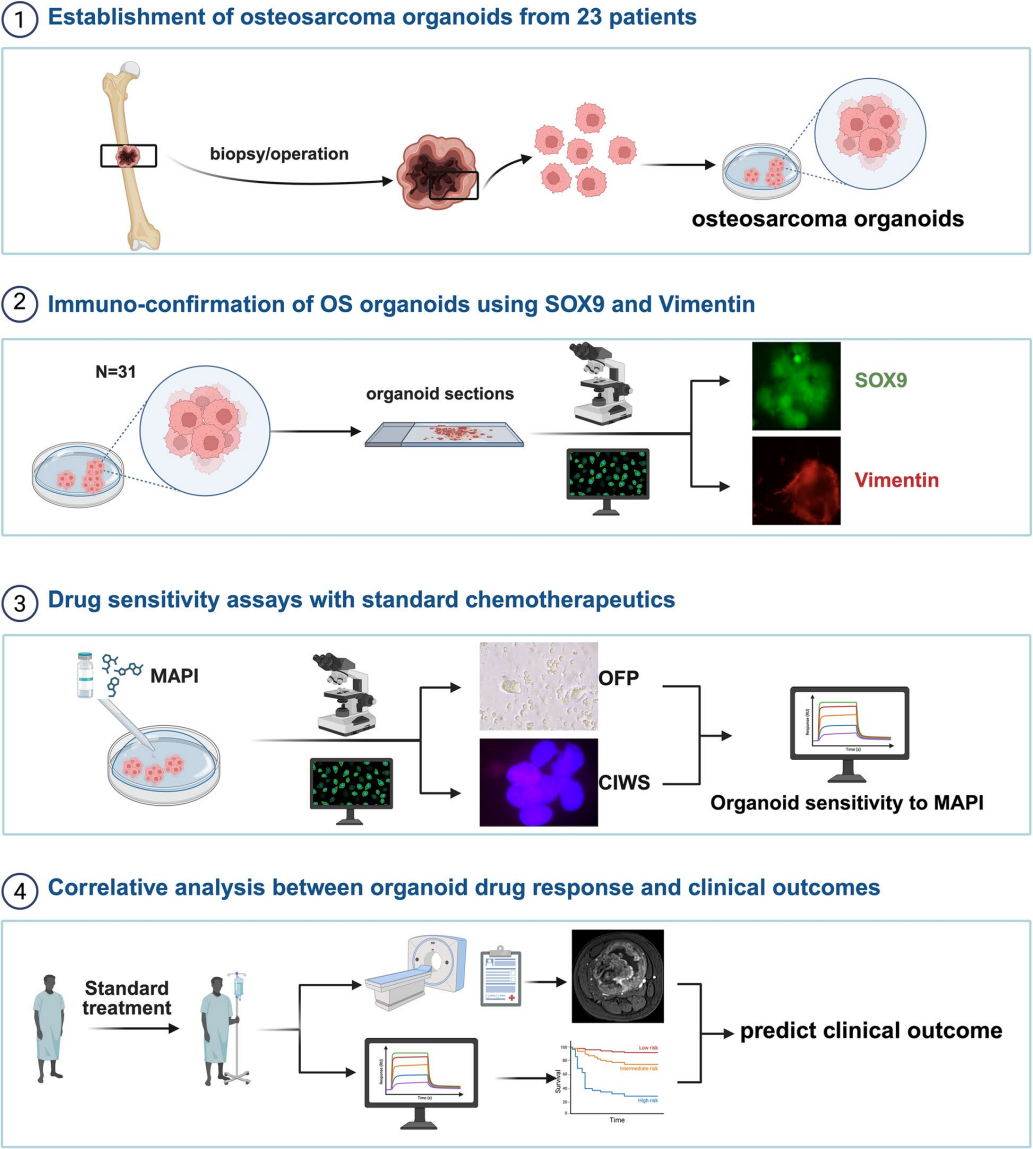
Surgical samples from 23 osteosarcoma patients were cultured into organoids. The patients’ response to MAPI treatment was analyzed via OFP/CIWS and compared with the patients’ clinical responses to validate the model

The demographic information of the enrolled patients is summarized in Table 1. A total of 23 patients were included (Table 1; Fig. 1). Male sex: 14/23 (60.9%); age, median (IQR): 18.0 (14.5–25.5) years; $\le$25 Years: 17/23 (73.9%). We obtained 31 tumor samples for downstream analyses (18 pre-NAT samples and 13 post-NAT samples), including 8 paired pre/post-NAT samples. PDOs were successfully established from 31 samples and composed the evaluable PDO analysis set for functional validation and drug-response testing.

**Table 1:**
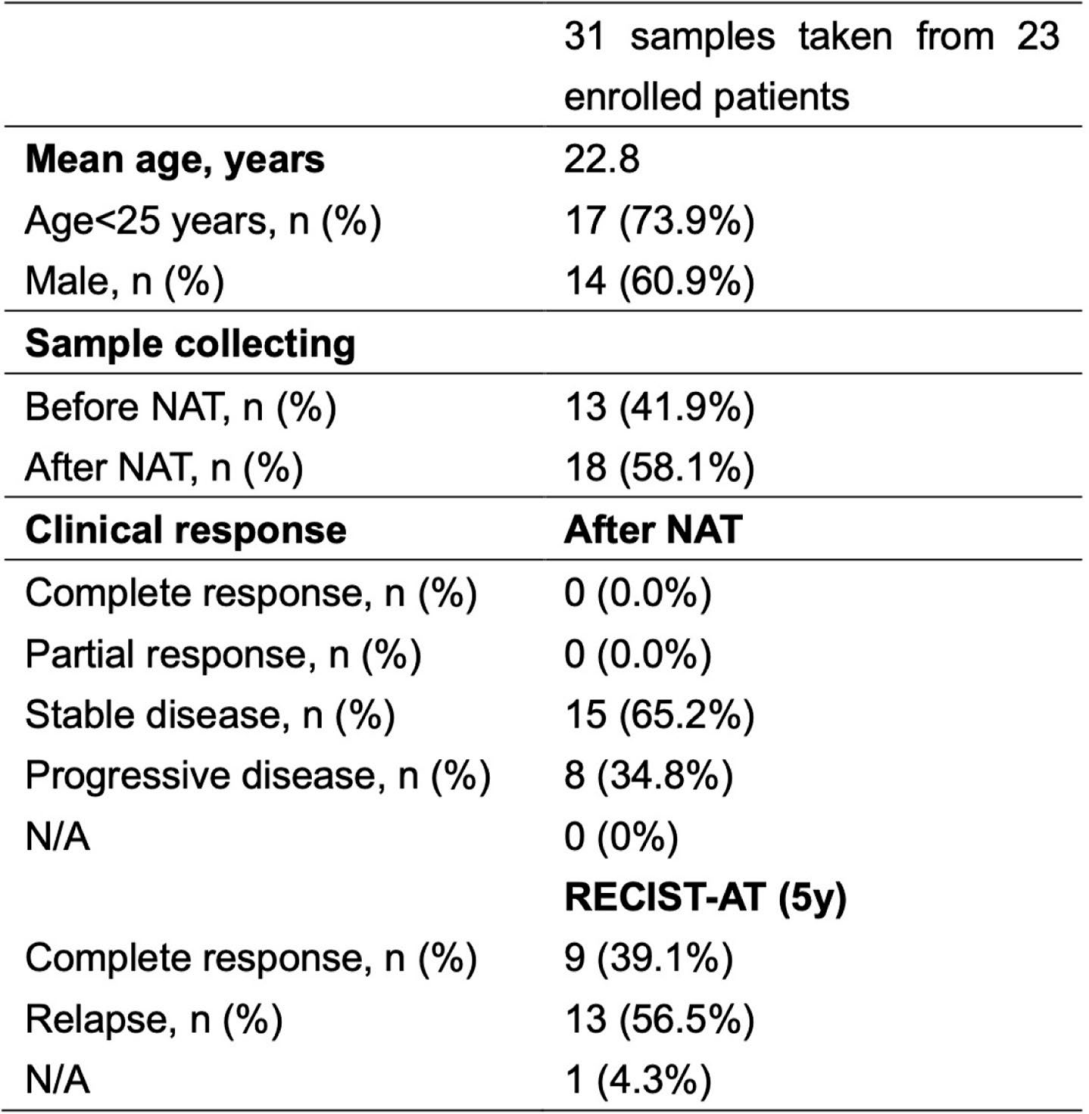
Osteosarcoma organaoid patient demographics

*N/A* not applicable, *RECIST-AT (5y)* overall survival at 5 yeaars after curative-intent surgery

**Successful culture of OSOs from pre-NA** **T** **osteosarcoma biopsies**

**Fig. 2:**
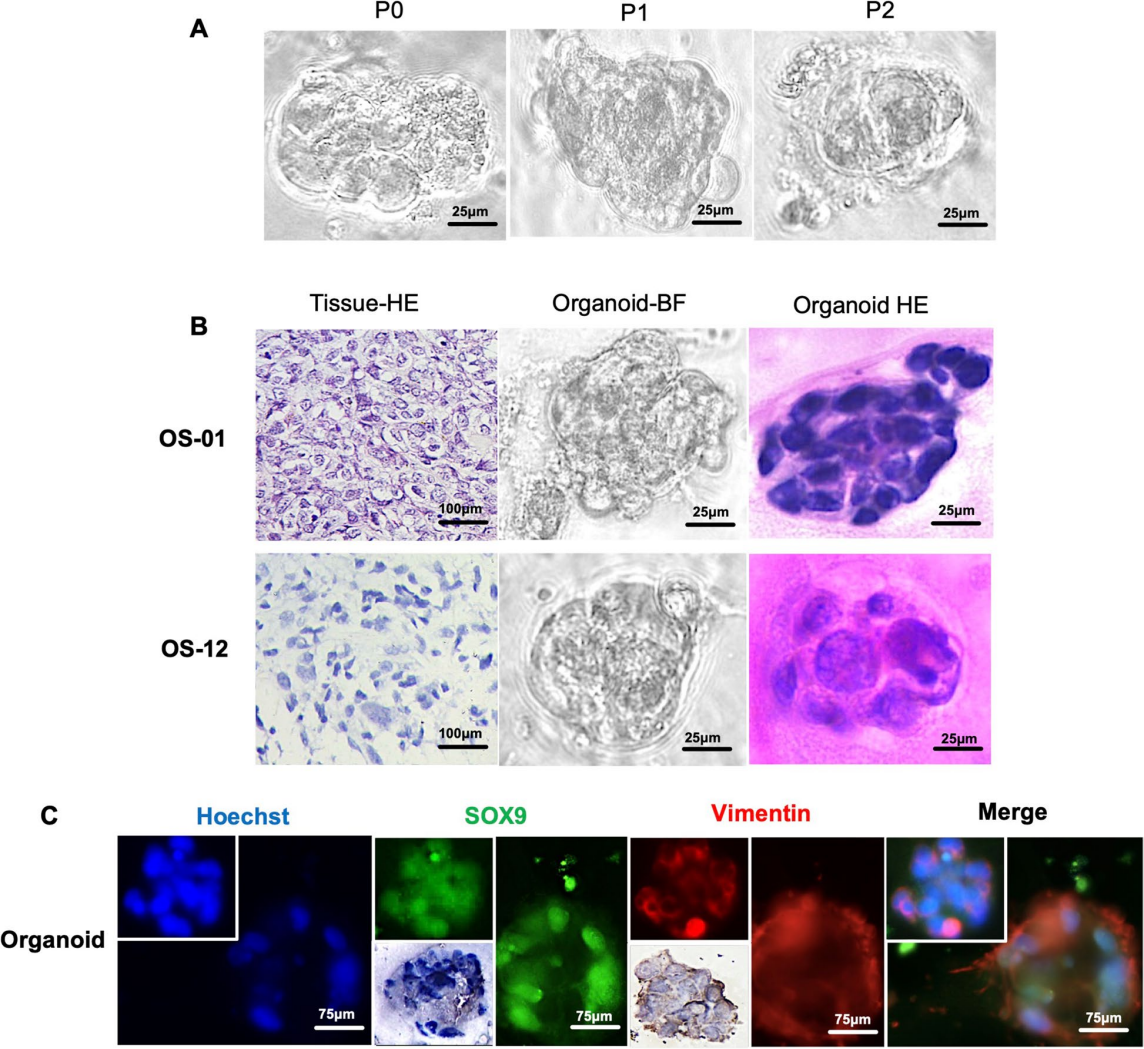
PDO establishment and identification. **A** Bright field image of the PDO (40x) after establishment and passage 2 times. **B** Pathomorphology of the original OS tumor H&E image, which corresponds to the organoid bright field (40x) and H&E staining (40x). **C** IFC of the OS-specific markers SOX9 (green) and vimentin (red) in the organoids (40x)

Tumor tissues were collected at the time of biopsy and surgical resection for each patient and processed to establish patient-derived osteosarcoma organoids (PDOs) following the protocol described previously (Table 2) [24]. Organoid establishment was considered successful when the cultures demonstrated sustained growth and could be serially passaged following initial plating (Fig. 2A). All the specimens, including both original tumor tissues and derived organoids, were archived in our institutional biobank and utilized for downstream functional analyses. In total, osteosarcoma tissues from 23 patients were collected, with all patients having undergone neoadjuvant chemotherapy with the MAPI regimen (high-dose methotrexate, doxorubicin, cisplatin, and ifosfamide). Among the 31 tumor samples, 18 were obtained via biopsy, and 13 were obtained from surgical resection. Paired pre- and post-NAT samples were available for eight patients. All the organoids were successfully formed, as summarized in Table 2, as determined by the emergence of organoid structures within 1–3 passages of culture initiation. To characterize the histological features of the established organoids, hematoxylin and eosin (H&E) staining was performed to assess the distribution of tumor cells and extracellular matrix (ECM) components (Fig. 2B). Furthermore, immunofluorescent colabeling of SOX9 and vimentin—two hallmark markers of osteosarcoma—confirmed that the PDOs preserved key molecular characteristics of their parental tumors (Fig. 2C).

**Table 2:**
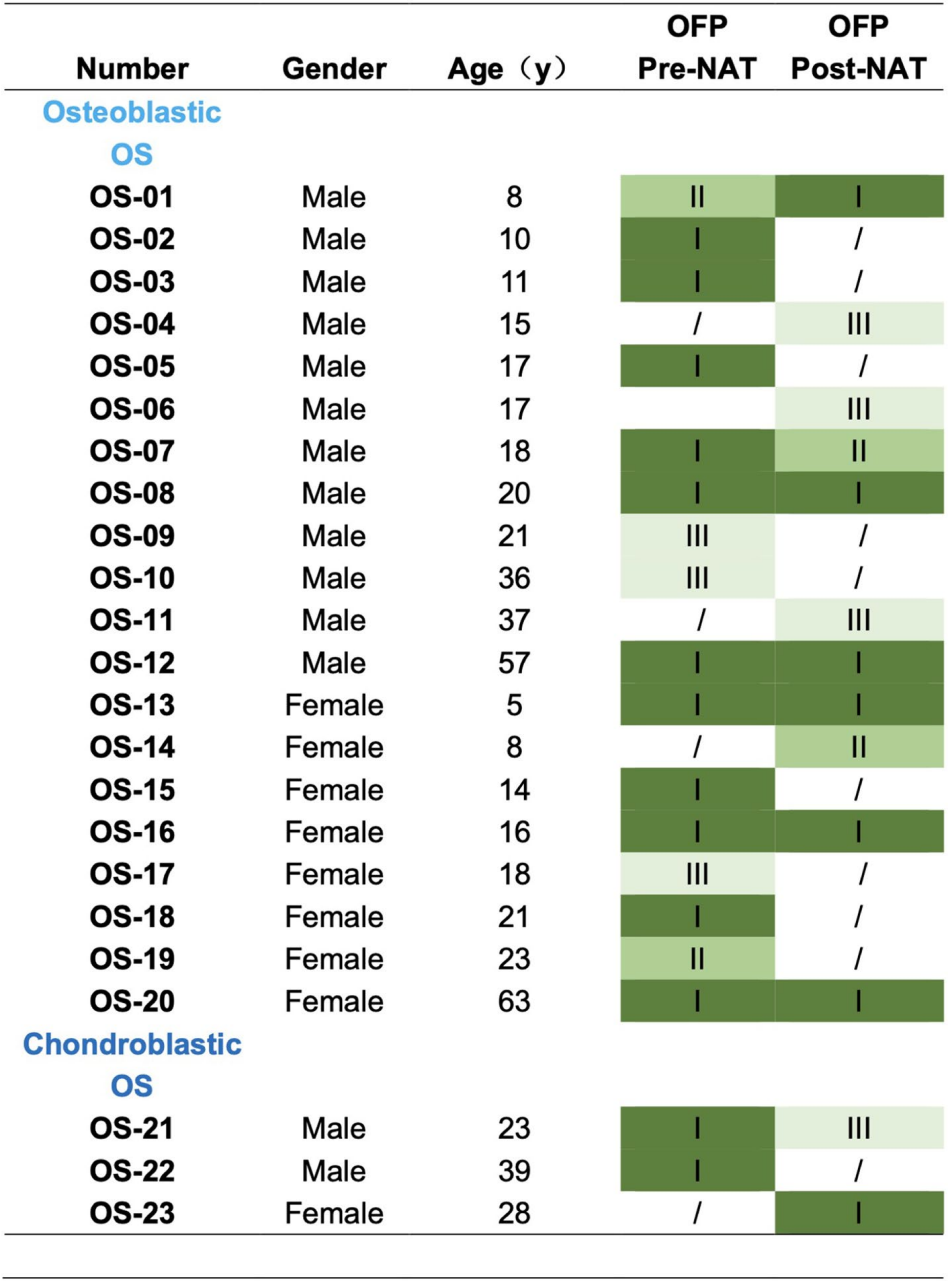
Statistics of organoid forming potential

*OS* osteosarcoma, *OFP* organoid forming potential

**Organoid Formation Potential Reflects Post-NAT Tumor Proliferation Dynamics**

**Fig. 3:**
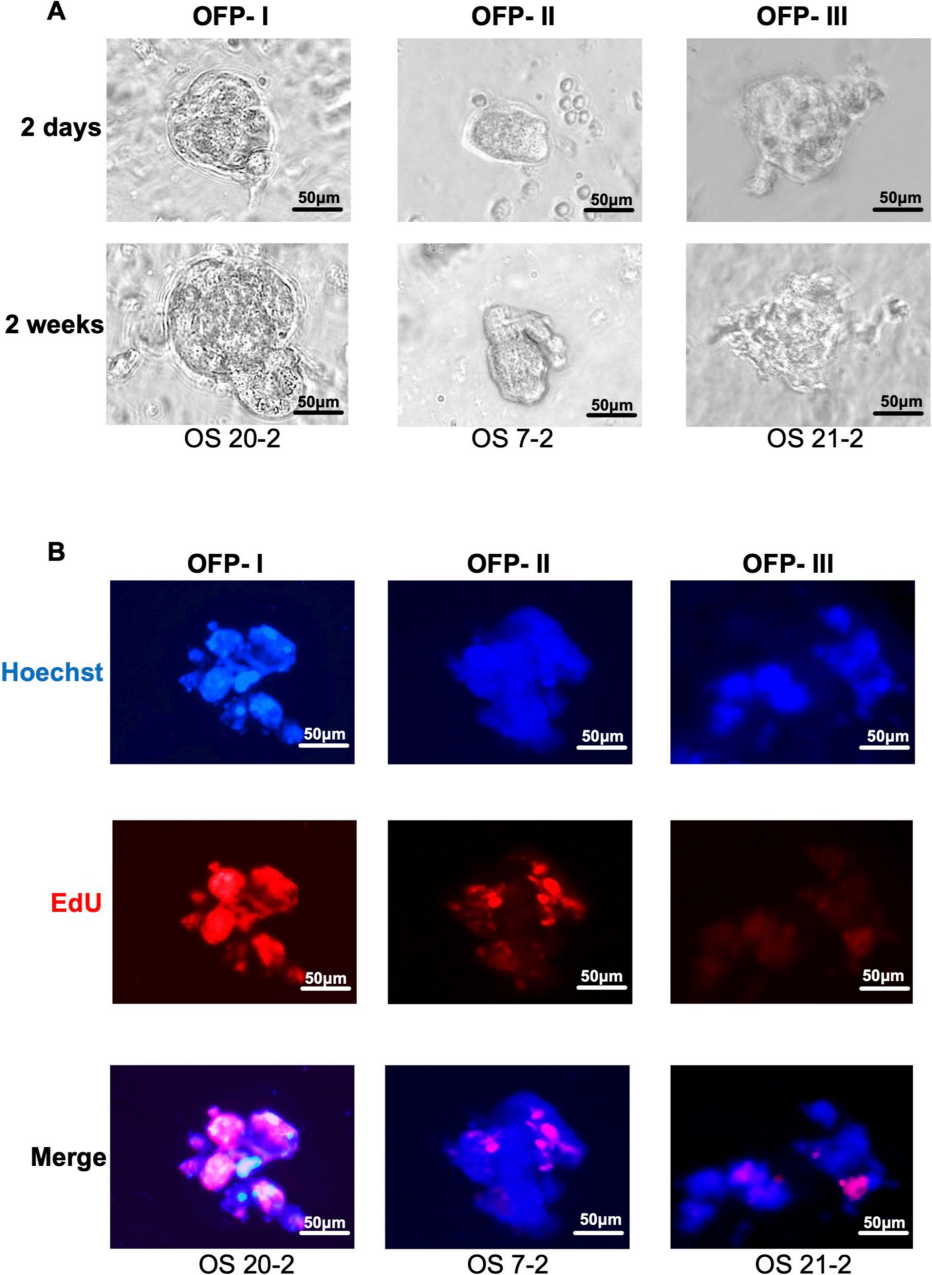
Organoid formation potential (OFP) grading for OS organoids. **A** Bright field images ($20\times$) of different OFP organoids of grades I-III at 2 days and 2 weeks. **B** EdU proliferation fluorescence staining of different OFP organoids (20x)

A total of 13 post-NAT osteosarcoma samples were subjected to patient-derived organoid (PDO) culture, including 8 paired samples with corresponding pre-NAT biopsies and 5 post-NAT-only samples. Organoid formation potential (OFP) was categorized into three levels—OFP-I (Robust-forming), OFP-II (Intermediate-forming), and OFP-III (Minimal/Nonforming)—on the basis of EdU fluorescence intensity and organoid size after three weeks of culture, reflecting the proliferative capacity of viable tumor cells within three-dimensional organoid structures (Fig. 3A-B; Table 2).

Among the 13 post-NAT samples, 7 (53.8%) presented high organoid formation potential (OFP-I), 2 (15.4%) presented moderate potential (OFP-II), and 4 (30.8%) presented low potential (OFP-III). Among the 8 patients with paired pre- and post-NAT specimens, 5 maintained stable OFP grades, whereas 3 showed dynamic shifts. In 1 patient (OS01) exhibited an increase in OFP from grade II to I following NAT and experienced an unfavorable clinical course, with a disease-free survival (DFS) of only 3 months and an overall survival (OS) of 38 months. Notably, patient OS07 exhibited a reduction in OFP from grade I to II post-NAT and achieved excellent long-term outcomes (DFS = 60 months, OS = 60 months). This observation suggests that decreased posttreatment organoid-forming capacity may reflect diminished tumor viability and be correlated with improved prognosis.

**Fig. 4:**
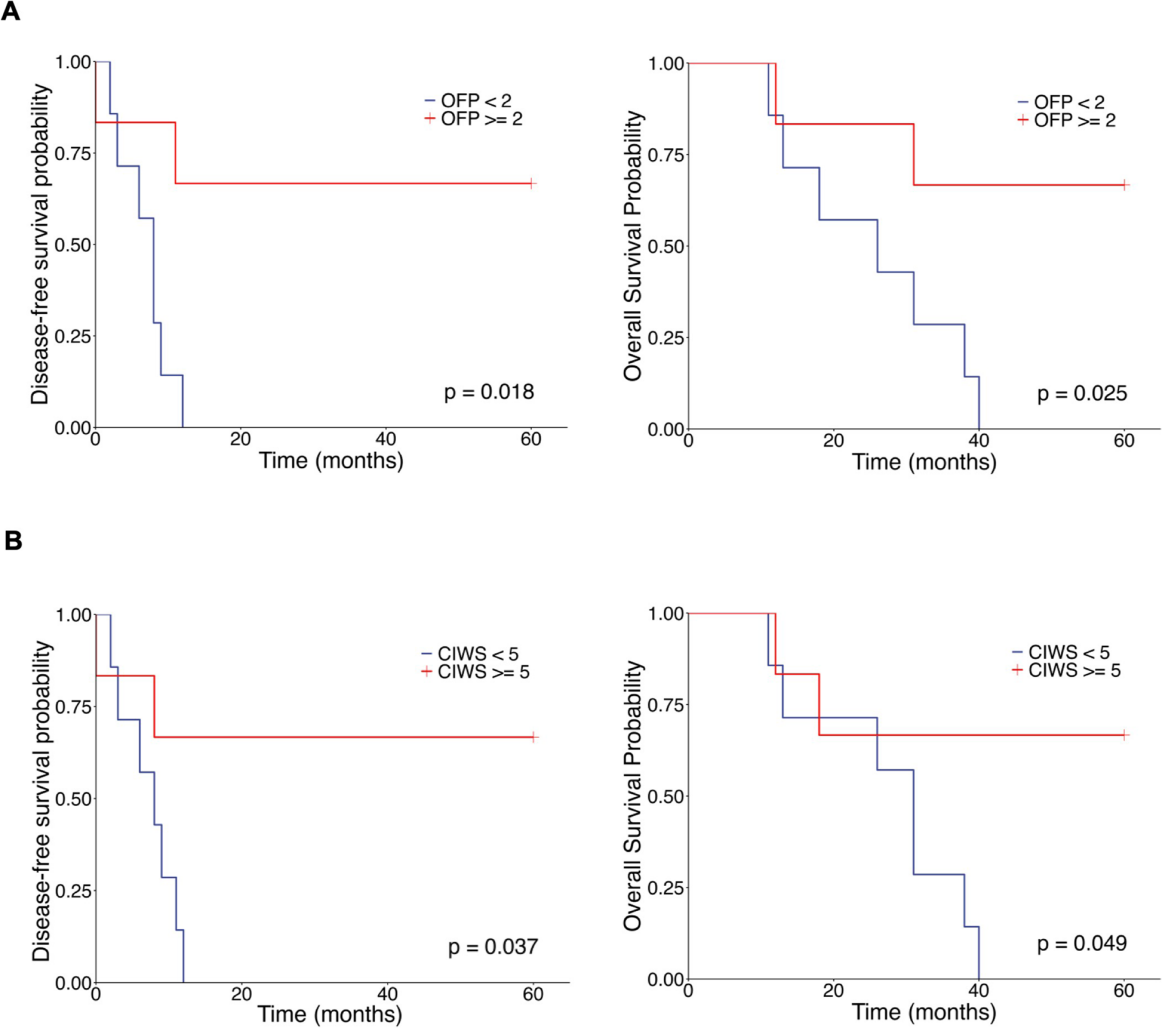
Kaplan–Meier survival curves stratified by organoid formation potential (OFP) and the cell inhibition rate score (CIWS). **A** Patients in the OFP-II+III group had markedly longer DFS and OS than those in the OFP-I group (mean DFS 41.8 vs 6.9 months, $\Delta =+34.9$; mean OS 47.2 vs 25.3 months, $\Delta =+21.9$; log-rank: DFS *P*=0.018; OS *P*=0.025). **B** Patients in the sensitive group (CIWS $\ge$ 5) had markedly longer DFS and OS than those in the non-sensitive group (CIWS $< 5$) (mean DFS 41.3 vs 7.3 months, $\Delta =+34.0$; mean OS 45.0 vs 27.1 months, $\Delta =+17.9$; log-rank: DFS *P*=0.037; OS *P*=0.049)

After OFP-I was designed as the high–growth-potential group and OFP-II/III as the low–growth-potential group (see **Methods-Accuracy calculation**), Kaplan–Meier (KM) analyses revealed superior outcomes in the low–growth-potential cohort (Fig. 4A): for DFS, the low–growth-potential group presented a lower risk of progression than did the high–growth-potential group did (Cox HR = 0.17, 95%CI 2–75; log-rank *P*= 0.018); for OS, the pattern was similar (HR = 0.19, 95% CI 3–83; log-rank *P*=0.025). These findings suggest that attenuated posttreatment organoid-forming capacity aligns with reduced tumor viability and improved prognosis.

**Chemosensitivity Shifts in Osteosarcoma Organoids Following Neoadjuvant Therapy**

**Fig. 5:**
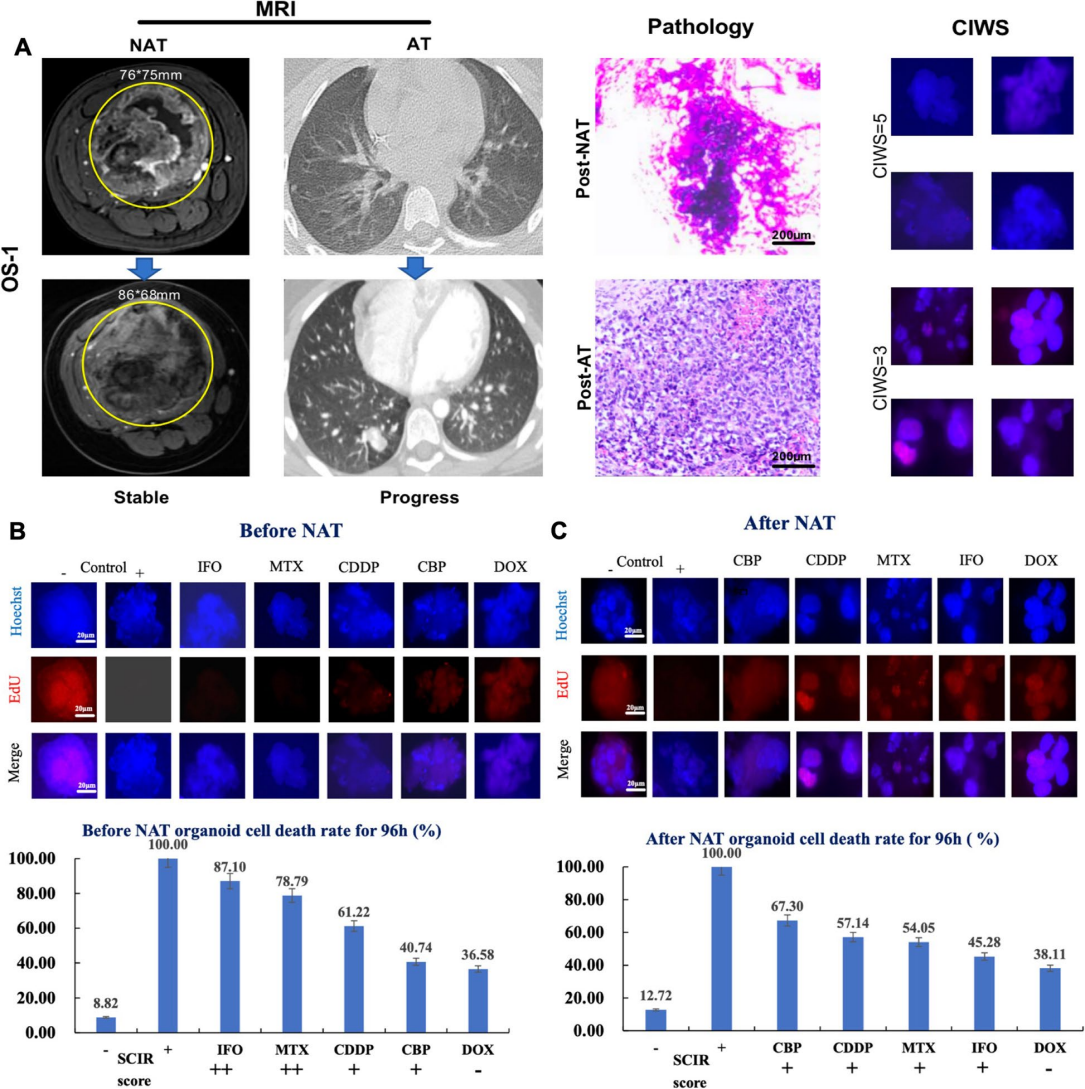
Dynamic drug sensitivity prediction in patient OS01. **A** Pre- and post-NAT MRI scans showing decreased enhancement but stable tumor size. Postoperative pathology revealed $>90$% necrosis. Follow-up chest CT demonstrated progressive bilateral lung metastases. **B** Pre-NAT PDO showed high sensitivity to MAPI chemotherapy (CIWS = 5+). **C** Post-NAT PDO demonstrated reduced sensitivity (CIWS = 3+), indicating acquired resistance. PDO predictions were consistent with both the histological response and clinical relapse. Abbreviations: “-” : negative control, “+” : positive control, DOX doxorubicin, CDDP cisplatinum, MTX methotrexate, IFO ifosfamide, CBP carboplatin

High-throughput drug screening was conducted on osteosarcoma organoids derived from biopsies and surgical specimens from 23 patients via first-line chemotherapeutic agents (doxorubicin, cisplatin, methotrexate, and ifosfamide) and the investigational agent carboplatin. Drug responses were quantified as the standard cell inhibition rate (SCIR), which was calculated as the difference between inhibition rates in drug-treated and control samples. The responses were stratified into four categories according to the SCIR values: – ($\textrm{SCIR} < 30\%$), + ($30\% \le \textrm{SCIR} < 60\%$), ++ ($60\% \le \textrm{SCIR} < 80\%$), and +++ ($\textrm{SCIR} \ge 80\%$) (Table 3). The cumulative SCIR score for the MAPI regimen served as a predictive marker, with total scores $<5+$ indicating resistance and scores $\ge 5+$ indicating sensitivity. Among eight patients with paired pre- and post-NAT organoids, CIWS dynamics were generally consistent with RECIST. The three-level directional concordance (improved/stable/worsened) reached 75.0% (6/8), with Cohen’s $\upkappa = 0.53$. When treated as continuous variables, $\Delta$CIWS correlated with $-\Delta$RECIST (indicating concordant improvement or worsening), with Spearman $\rho = 0.682$ ($\textit{P} = 0.063$) and Kendall’s $\tau$-b $= 0.618$ ($\textit{P} = 0.056$). These findings suggest a moderate-to-strong concordant trend, albeit with borderline significance given the limited sample size. A representative case, patient OS01, exemplifies the dynamic shift in chemosensitivity and the predictive accuracy of PDO-based cumulative inhibition-weighted score (CIWS) profiling. As shown in Fig. 5A, pre-NAT contrast-enhanced MR images revealed a hypervascular osteosarcoma lesion in the right femur measuring approximately 76 $\times$ 75 mm. Following NAT, the lesion exhibited reduced enhancement but remained stable in size, which is consistent with radiologically stable disease. Histopathological analysis of the resected specimen revealed $>90$% tumor necrosis, indicating a strong pathological response to NAT. Correspondingly, the pre-NAT PDOs demonstrated a CIWS of $5+$ (Fig. 5B), indicating sensitivity to MAPI chemotherapy. However, post-NAT PDOs presented a markedly reduced CIWS of $3+$ (Fig. 5C), which is consistent with acquired chemoresistance. This shift in ex vivo drug sensitivity accurately foreshadows the patient’s clinical course: despite the initial response, OS01 patients experienced early pulmonary metastases after adjuvant chemotherapy. Subsequent CT scans revealed progressive, multifocal pulmonary nodules (Fig. 5A), indicating posttreatment disease progression.

**Table 3:**
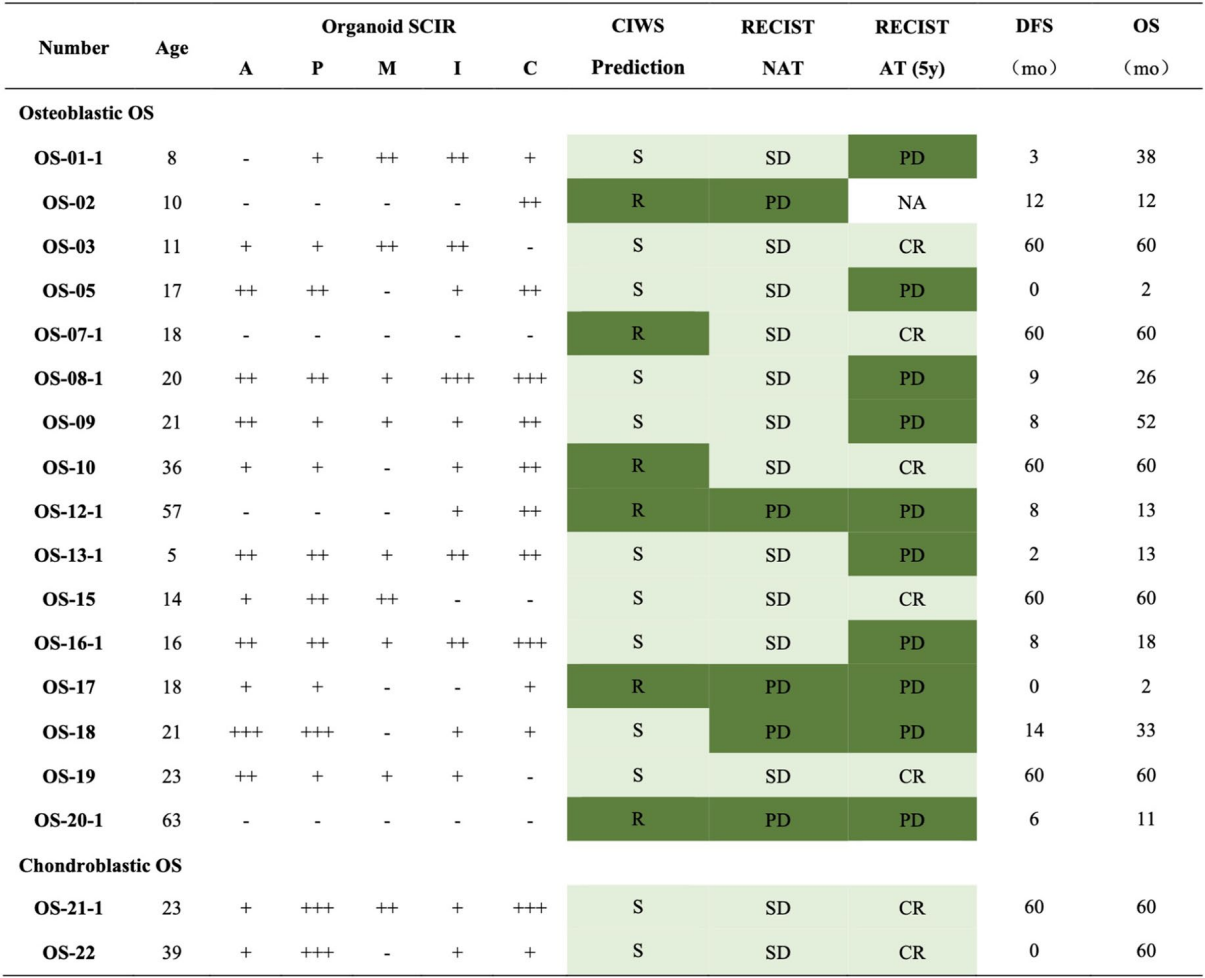
Organoid culture before neoadjuvant therapy

Cooperation between organoid drug prediction and clinical evaluation before neoadjuvant chemotherapy. Drugs: A doxorubicin, P platinum, M methotrexate, I ifosfamide, C carboplatin. CIWS prediction: cumulative inhibition-weighted score from PDO sensitivily to multiple agents. Symbols: “–”: not effective (SCIR $<30$%); “+”: possibly effective (SCIR $\ge 30$% and $<60$%); “++”: probably efective (SCIR $\ge 60$% and $<80$%); “+++”: highly effective (SCIR $\ge 80$%); Sensitively (S) = CIWS $\geqslant 5$ +; resistance (R) = CIWS $<5$+. RECIST NAT: radiologic responce per RECIST after completion of NAT. RECIST AT (5y): overall suvival at 5 years after curative-intent surgery

Abbreviations: *PD* progressive disease, *SD* stable disease, *CR* complete response, *DFS* disease-free survival, *OS* overall survival, *mo* months

These observations closely align with clinical findings, where most patients maintain stable drug sensitivity, and a minority acquire resistance during treatment. The SCIR-based organoid assay thus offers a robust, quantitative platform for assessing patient-specific drug responses, underscoring its value in guiding personalized osteosarcoma therapy.

**Pre-NAT Organoid Chemosensitivity Correlates with Radiologic and Long-Term Outcomes in Osteosarcoma**

Patient-derived osteosarcoma organoids (PDOs), generated from preneoadjuvant therapy (NAT) biopsies and surgical specimens from 23 patients, were subjected to high-throughput drug sensitivity screening. Chemosensitivity was quantified via the cumulative inhibition-weighted score (CIWS), which was calculated as the sum of standard cell inhibition rates (SCIRs) for four first-line chemotherapeutic agents comprising the MAPI regimen (methotrexate, doxorubicin, cisplatin, and ifosfamide). On the basis of the CIWS threshold, the organoids were classified as sensitive (CIWS $\ge$ 5+) or resistant (CIWS $<5$+). This integrated metric provides a robust, quantitative assessment of each organoid’s overall response to standard chemotherapy, enabling the prediction of clinical sensitivity and resistance.

**Fig. 6:**
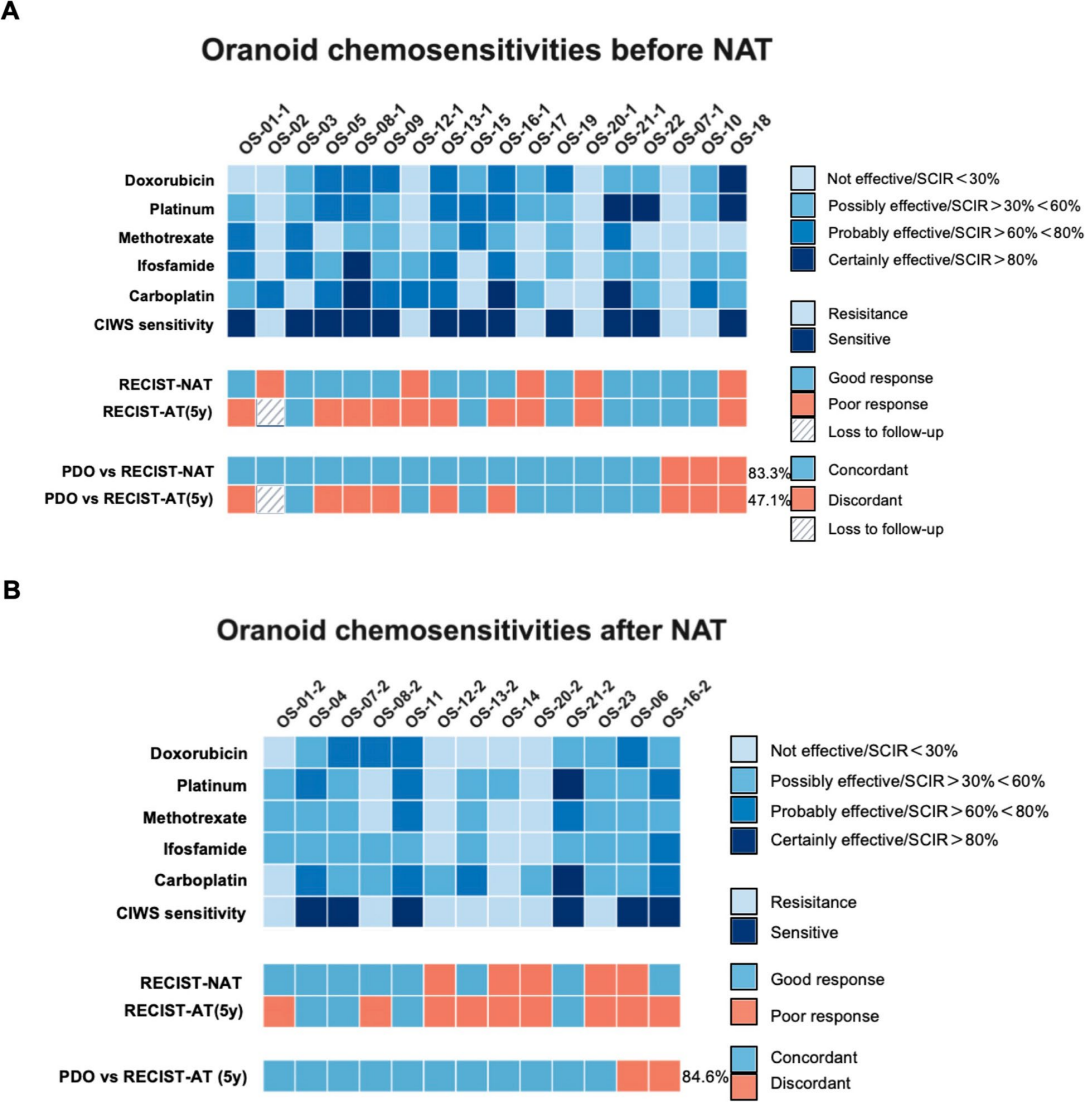
Concordance of PDO chemosensitivity with clinical response and prognosis. **A** Pre-NAT and **B** post-NAT heatmaps show drug-specific SCIR tiers ($<30$, 30–$<60$, 60–$<80$, $>=80$) and the composite CIWS across MAPI (“CIWS sensitivity”; sensitive $>=5$+, resistant $<=4$+). NAT-RECIST denotes RECIST 1.1 immediately after NAT; AT-RECIST (5y) denotes RECIST 1.1 at the 5-year follow-up after curative-intent surgery in patients who completed NAT, surgery, and adjuvant therapy; both binarized as responder (CR/PR/SD) vs non-responder (PD). Rows “PDO vs NAT-RECIST” and “PDO vs AT-RECIST (5y)” report concordance between PDO predictions (CIWS Sensitivity) and these labels (accuracy 83.3% and 84.6%, respectively). Abbreviations: PDO, patient-derived organoid; NAT, neoadjuvant therapy; AT, adjuvant therapy; SCIR, standard cell-inhibition rate; CIWS, cell-inhibition weighted score; RECIST, Response Evaluation Criteria in Solid Tumors; MAPI, methotrexate+doxorubicin+cisplatin+ifosfamide

Among 18 paired cases with pre-NAT organoid predictions and post-NAT radiologic assessments, CIWS-based drug-response testing (cutoff: CIWS $\ge 5+$ as predicted SD, $<5$+ as predicted PD) demonstrated high concordance with RECIST outcomes, yielding an overall accuracy of 83.3% (15/18; 95% CI 58.6–96.4; exact binomial *P*=0.004). Using PD (nonresponse) as the positive class, the sensitivity was 80.0% (4/5; 95% CI, 28.4–99.5), while specificity for SD (response) was 84.6% (11/13; 95% CI, 54.6–98.1). The PPV for PD was 66.7% (4/6; 95% CI, 22.3–95.7), and the NPV for SD was 91.7% (11/12; 95% CI, 61.5–99.8). Agreement was moderate (Cohen’s $\upkappa = 0.609$), and the exact McNemar test (*P* = 1.000; discordant pairs b = 1, c = 2) indicated no systematic bias (Fig. 6A, Table 3). Collectively, these findings support the utility of pre-NAT PDO assays quantified by CIWS in predicting post-NAT radiologic outcomes.

**Predictive** **power** **of** **post-NAT-PDO chemosensitivity for OS prognosis**

To comprehensively assess the prognostic value of postneoadjuvant therapy (NAT) chemosensitivity in osteosarcoma organoids (PDOs), we calculated a cumulative inhibition-weighted score (CIWS) for each post-NAT PDO. CIWS was defined as the sum of the standard cell inhibition rates (SCIRs) for four first-line chemotherapeutic agents—methotrexate, doxorubicin, cisplatin, and ifosfamide (MAPI regimen)—providing an integrated and quantitative measure of drug efficacy in the organoids. Among the 13 post-NAT PDOs tested, 6 were classified as CIWS-predicted sensitive PDOs, and 7 were classified as resistant PDOs. Using 5-year RECIST as the reference (CR=FAV; PD=ADV), post-NAT CIWS–based stratification correctly classified 11/13 patients (accuracy 84.6%; 95% CI 54.6–98.1; exact binomial *P*=0.011). The sensitivity for adverse outcomes was 77.8% (7/9; 95% CI 40.0–97.2), the specificity for favorable outcomes was 100% (4/4; 95% CI 39.8–100), the agreement was substantial (Cohen’s $\upkappa =0.68$), and the exact McNemar test revealed no directional misclassification bias (*P*=0.50) (Fig. 6B, Table 4). Compared with post-NAT CIWS stratification (Acc 84.6% [11/13; 95% CI 54.6–98.1]; Sens 77.8%; Spec 100%; $\upkappa =0.68$), pre-NAT RECIST predicted posttreatment RECIST less accurately (Acc 69.2% [9/13; 95% CI 38.6–90.9]; Sens 55.6%; Spec 100%; $\upkappa =0.43$). For paired correctness, CIWS-only correct = 3 versus RECIST-only correct = 1, a nonsignificant difference (exact McNemar *P*=0.625). CIWS also showed higher balanced accuracy (88.9% vs 77.8%) (Fig. 6B). These data indicate that CIWS offers a more precise and comprehensive prediction of posttreatment outcomes than do conventional imaging modalities.

**Table 4:**
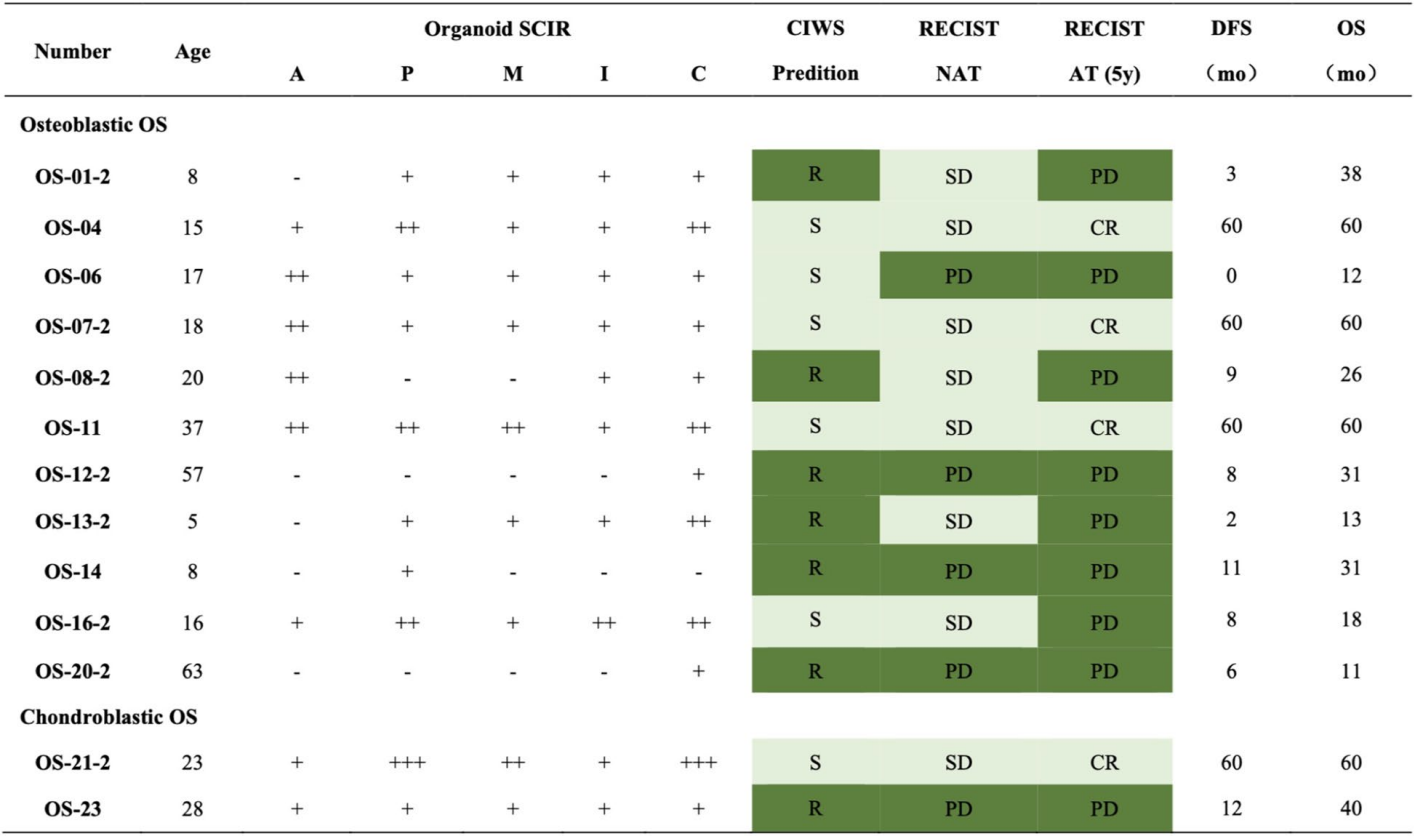
Organoid culture after neoadjuvant therapy

Cooperation between organoid drug prediction and clinical evaluation after neoadjuvant chemotherapy. Drugs: A doxorubicin, P cisplatinum, M methotrexate, I ifosfamide, C carboplatin. CIWS prediction: cumulative inhibition-weighted score from PDO sensitivily to multiple agents. Symbols: “–”: not effective (SCIR $<30$%); “+”: possibly effective (SCIR $\ge 30$% and $<60$%); “++”: probably efective (SCIR $\ge 60$% and $<80$%); “+++”: highly effective (SCIR $\ge 80$%); Sensitively (S) = CIWS $\geqslant 5$ +; resistance (R) = CIWS $<5$+. RECIST NAT: radiologic responce per RECIST after completion of NAT. RECIST AT (5y): overall suvival at 5 years after curative-intent surgery

Abbreviations: *PD* progressive disease, *SD* stable disease, *CR* complete response, *DFS* disease-free survival, *OS* overall survival, *mo* months

Importantly, Kaplan–Meier survival analyses further validated the prognostic value of CIWS-guided drug sensitivity profiling. Patients whose PDOs were predicted to be sensitive to the MAPI regimen exhibited statistically significant improvements in disease-free survival (DFS) and overall survival (OS) compared with those whose PDOs were classified as resistant (Fig. 4B). Post-NAT PDOs were dichotomized by CIWS (sensitive $\ge$5+, resistant <5+; see Methods-Accuracy calculation). Kaplan–Meier analyses indicated superior outcomes in the CIWS-sensitive cohort (Fig. 4B). For DFS, the CIWS-sensitive group had a lower risk of progression than the CIWS-resistant group (Cox HR 0.21; 95% CI 4–105; log-rank *P*=0.037). For OS, a similar pattern was observed (HR 0.23; 95% CI 5–107; log-rank *P*=0.049). These data Link higher CIWS-defined chemosensitivity with reduced posttreatment risk and improved prognosis. Because the Wald 95% CIs marginally include 1.0—reflecting the small number of events—the effect sizes should be interpreted cautiously and validated prospectively.

Collectively, these findings underscore the robustness of post-NAT PDO drug sensitivity assays, quantified via CIWS, in accurately predicting patient prognosis. By integrating PDO-derived chemosensitivity data into clinical decision-making, this approach holds promise to surpass conventional imaging assessments in identifying patients likely to benefit from long-term disease control versus those at greater risk of recurrence.

## Discussion

Osteosarcoma (OS) is a highly heterogeneous malignancy with variable responses to treatment, and effective methods to predict therapeutic efficacy remain limited. Neoadjuvant chemotherapy (NAT) is the standard preoperative strategy for OS, aiming to reduce recurrence rates and improve disease-free survival (DFS) [25]. However, its efficacy evaluation predominantly relies on imaging and pathological criteria, which are often delayed and lack sufficient accuracy [26, 27]. In this study, we established a patient-derived osteosarcoma organoid (PDO) platform that integrates in vitro drug sensitivity assays with clinical outcomes (RECIST criteria and long-term prognosis). Our results demonstrated that the PDO-based drug sensitivity prediction achieved an accuracy of 84.6% (11/13), which was significantly greater than that of the conventional RECIST standard (69.2%, 9/13), highlighting its superiority in precisely predicting the NAT response.

To establish and evaluate the drug sensitivity of PDOs, we propose an OFP grading system that reflects the proliferative capacity of residual tumors. Our findings revealed that OFP is closely associated with clinical outcomes and long-term prognosis, with lower OFP levels (indicating loss of formation potential) correlating with improved DFS and overall survival (OS) [28]. Notably, in the eight paired samples collected before and after NAT, PDOs revealed dynamic changes in acquired resistance (e.g., OFP shifting from low to high) and showed high concordance between in vitro drug sensitivity and long-term clinical outcomes.

Furthermore, we applied a combined inhibitory weight score (CIWS) system, integrating the effects of four first-line anti-OS drugs (methotrexate, doxorubicin, cisplatin, and ifosfamide) to better simulate clinical sequential chemotherapy. Compared with previous studies focused solely on single-drug sensitivity [29], CIWS achieved 84.6% accuracy for predicting 5-year prognosis in the post-NAT cohort ($n=13$) and 83.3% accuracy for anticipating preoperative chemotherapy response changes in the pre-NAT cohort ($n=18$), as assessed by RECIST 1.1. These findings indicate that PDOs may aid in predicting neoadjuvant therapy (NAT) efficacy and long-term prognosis, which aligns with findings from PDO studies in colorectal cancer [30], gynecologic tumors [31], and pancreatic ductal adenocarcinoma [32]. Importantly, PDO testing can not only predict sensitivity to standard NAT regimens but also suggest alternative drugs (e.g., carboplatin) for resistant patients, potentially reducing unnecessary chemotherapy-induced side effects and supporting personalized treatment strategies [33].

Despite these promising findings, the PDO model has certain limitations. First, the sample size in this study was relatively small (23 cases), and extended follow-up is needed to confirm its long-term predictive value. Second, the limited volume of biopsy samples may not fully represent tumor heterogeneity, and the interval between biopsy and follow-up (five years) may allow for genetic mutations and resistance evolution [34]. Additionally, while the PDO culture system can mimic certain aspects of the tumor microenvironment, it lacks complex interactions with the immune system and angiogenesis [35]. Future studies should integrate multiomics analyses (genomics, transcriptomics, proteomics) and immune-related research to increase the predictive ability and clinical applicability of PDOs [36]. Furthermore, technological advancements and regulatory support are essential to integrate PDO testing into clinical workflows, ensuring widespread application and cost-effectiveness.

This study explores a dual-dimension functional readout—CIWS and OFP—that may help narrow the temporal gap inherent to imaging for detecting early non-response and acquired resistance. Relative to RECIST, CIWS appeared to provide higher prognostic accuracy in post-NAT specimens and to reflect within-patient shifts in chemosensitivity across paired samples during therapy, suggesting that PDO testing could offer decision-informing evidence for real-time treatment triage. On this basis, we outline a pragmatic clinical pathway: in the pre-NAT setting, patients with CIWS $\ge 5$ might reasonably continue or intensify standard MAPI, whereas those with CIWS $< 5$ could be considered for dose/sequence optimization, alternative agents (e.g., carboplatin evaluated here), or clinical trial enrollment; in the post-NAT setting, when OFP-I co-occurs with CIWS $< 5$, closer surveillance and earlier intervention may be warranted. We acknowledge that the CIWS and OFP thresholds were derived through internal optimization with bootstrap resampling and therefore require external calibration and prospective evaluation in independent cohorts. In parallel, the current PDO system may not fully recapitulate in-vivo pharmacokinetics/pharmacodynamics (PK/PD) and the bone-specific tumor microenvironment; future iterations—such as exposure to active metabolites, organ-on-chip perfusion, and immune/stromal co-culture—are planned to enhance translatability.

In summary, osteosarcoma PDOs offer a precise approach for predicting the NAT response and serve as powerful tools for assessing long-term prognosis, identifying resistance evolution, and formulating personalized treatment strategies. With advancements in PDO culture and testing technologies, their application potential in OS and other solid tumors will continue to expand, contributing to the development of functional precision oncology [37]. We anticipate that future multicenter validations, integration with molecular biomarkers, and standardized protocols will facilitate the widespread clinical implementation of PDO technology.

## Conclusion

This study established a patient-derived organoid (PDO) platform to predict neoadjuvant chemotherapy (NAT) response and long-term outcomes in osteosarcoma. CIWS-based profiling of post-NAT specimens achieved higher predictive accuracy than RECIST (84.6% (11/13) vs 69.2% (9/13)) and revealed acquired-resistance dynamics and potential alternative agents. Assays were completed within a clinically actionable window ($\le$2 months). Together with the organoid-forming potential (OFP) grade, CIWS provides complementary functional information beyond RECIST and pathological necrosis to support risk-adapted therapy and surveillance. Because CIWS/OFP cut-offs were internally derived, external calibration and prospective multicentre validation are required before routine implementation. Overall, PDO testing serves as a practical bridge to functional precision oncology in osteosarcoma, informing real-time treatment decisions.

## Data Availability

No datasets were generated or analysed during the current study.
